# FAIR4Health: Findable, Accessible, Interoperable and Reusable data to foster Health Research

**DOI:** 10.12688/openreseurope.14349.1

**Published:** 2022-03-09

**Authors:** Celia Alvarez-Romero, Alicia Martínez-García, A. Anil Sinaci, Mert Gencturk, Eva Méndez, Tony Hernández-Pérez, Rosa Liperoti, Carmen Angioletti, Matthias Löbe, Nagarajan Ganapathy, Thomas M. Deserno, Marta Almada, Elisio Costa, Catherine Chronaki, Giorgio Cangioli, Ronald Cornet, Beatriz Poblador-Plou, Jonás Carmona-Pírez, Antonio Gimeno-Miguel, Antonio Poncel-Falcó, Alexandra Prados-Torres, Tomi Kovacevic, Bojan Zaric, Darijo Bokan, Sanja Hromis, Jelena Djekic Malbasa, Carlos Rapallo Fernández, Teresa Velázquez Fernández, Jessica Rochat, Christophe Gaudet-Blavignac, Christian Lovis, Patrick Weber, Miriam Quintero, Manuel M. Perez-Perez, Kevin Ashley, Laurence Horton, Carlos Luis Parra Calderón

**Affiliations:** 1 Group of Research and Innovation in Biomedical Informatics, Biomedical Engineering and Health Economy, Institute of Biomedicine of Seville, IBiS / Virgen del Rocío University Hospital / CSIC / University of Seville, Seville, 41013, Spain; 2 SRDC Software Research Development and Consultancy Corporation, Ankara, 06800, Turkey; 3 Dept. of Library & Inf Sci. Universidad Carlos III de Madrid, Getafe, 28903, Spain; 4 Department of Geriatric and Orthopedic Sciences, Catholic University of Sacred Heart, Roma, 00168, Italy; 5 Institute for Medical Informatics (IMISE), University of Leipzig, Leipzig, 04107, Germany; 6 PLRI Institute for Medical Informatics of TU Braunschweig and Hannover Medical School, Braunschweig, 38106, Germany; 7 Ucibio Requimte, Faculty of Pharmacy University of Porto. Porto4Ageing, Porto, 4050-313, Portugal; 8 HL7 Europe Foundation, Brussels, 1000, Belgium; 9 Amsterdam UMC, University of Amsterdam, Medical Informatics, Amsterdam Public Health, Amsterdam, 1105AZ, The Netherlands; 10 EpiChron Research Group, Aragon Health Sciences Institute (IACS), IIS Aragón, Miguel Servet University Hospital, Zaragoza, 50009, Spain; 11 EpiChron Research Group, Aragon Health Sciences Institute (IACS), IIS Aragón, Aragon Health Service, Zaragoza, 50009, Spain; 12 Medical Faculty University of Novi Sad, Novi Sad, 21000, Serbia; 13 Institute for Pulmonary Diseases of Vojvodina, Sremska Kamenica, 21204, Serbia; 14 J&A Garrigues, S.L.P., Seville, 41013, Spain; 15 University of Geneva and University hospitals of Geneva, Geneva, 1211, Switzerland; 16 Nice Computing SA Le Mont-sur-Lausanne, Le Mont-sur-Lausanne, 1052, Switzerland; 17 Atos Research and Innovation - ARI. Atos IT., Madrid, 28037, Spain; 18 Atos Research and Innovation - ARI. Atos Spain., Madrid, 28037, Spain; 19 Digital Curation Centre, University of Edinburgh, Argyle House, Edinburgh, EH3 9DR, UK; 20 Digital Curation Centre, University of Glasgow, Glasgow, G12 8QQ, UK

**Keywords:** FAIR principles, health research data management, HL7 FHIR, health data, data sharing, data reuse, health research, open science, privacy-preserving computing, machine learning.

## Abstract

Due to the nature of health data, its sharing and reuse for research are limited by ethical, legal and technical barriers. The FAIR4Health project facilitated and promoted the application of FAIR principles in health research data, derived from the publicly funded health research initiatives to make them Findable, Accessible, Interoperable, and Reusable (FAIR). To confirm the feasibility of the FAIR4Health solution, we performed two pathfinder case studies to carry out federated machine learning algorithms on FAIRified datasets from five health research organizations. The case studies demonstrated the potential impact of the developed FAIR4Health solution on health outcomes and social care research. Finally, we promoted the FAIRified data to share and reuse in the European Union Health Research community, defining an effective EU-wide strategy for the use of FAIR principles in health research and preparing the ground for a roadmap for health research institutions to offer access to certified FAIR datasets.

This scientific report presents a general overview of the FAIR4Health solution: from the FAIRification workflow design to translate raw data/metadata to FAIR data/metadata in the health research domain to the FAIR4Health demonstrators’ performance.

## Plain language summary

Health research organizations work more and more with health data. The reuse of health data has significant benefits for society, both financially and for our well-being. However, there are significant barriers to data sharing, which our project has taken steps to overcome. The FAIR principles of Findability, Accessibility, Interoperability and Reusability are intended to influence such institutions to support collaborative use of data. FAIR4Health promotes the application of FAIR principles in health research data derived from public projects. FAIR4Health developed a workflow and tools to support the FAIR principles, and applied these to two case studies, extending across multiple health care sites, which confirmed feasibility.

## Introduction

One of the more significant challenges of data-intensive science is to facilitate the breakthrough of knowledge by assisting humans and machines in the discovery, access, integration, and analysis of task-appropriate scientific data and their associated algorithms and workflows, facilitating reproducibility of the research.

The FAIR guiding principles describe distinct considerations for contemporary data publishing environments with respect to supporting both manual and automated deposition, exploration, sharing, and reuse, describing a set of guiding principles to make data Findable, Accessible, Interoperable, and Reusable ^1^. Furthermore, the FAIR principles ensure that data are shared to enable and enhance reuse by humans and machines. Although FAIR emerged from a workshop for the life science community, the principles are intended to be applied to data and metadata from all disciplines.

Since their formal release via the FORCE11 community, FAIR principles have been adopted by several funders and governments worldwide. The European Commission data management guidelines were updated in 2017 to introduce the notion of FAIR. The European Open Science Cloud (EOSC) Declaration and recent EOSC Strategic Research and Innovation Agenda ( EOSC SRIA) both emphasise the central role of FAIR data.

In addition, it is essential to refer to the report issued by the European Union about the costs of NOT having FAIR data ^2^, whose main conclusions are that: i) the cost of NOT having FAIR data is approximately €10.2bn per year for the EU; ii) in addition, the open data economy suggests that the impact on innovation of FAIR could add another €16bn to the minimum cost estimated; and iii) that would make a total of at least €26.2bn per year.

A diverse range of research disciplines are adopting FAIR principles. Several groups have been assessing FAIR uptake to date and the challenges being encountered. In the same way, the FAIR4Health project, which has received funding from the European Union’s Horizon 2020 research and innovation programme under grant agreement No 824666, promotes the application of FAIR principles to health research data.

## Methods

First of all, we performed a comprehensive analysis of current barriers, facilitators and potential overcoming mechanisms in the EU to implement a FAIR data policy in health research institutions. Information from different perspectives (technical, ethical, security, legal, cultural, behavioral and economic) was gathered to generate guidelines providing an optimal strategy for implementing this policy in EU health research institutions. Concretely, a FAIR4Health public deliverable ^3^ provided an analytical overview of all the considerations addressed to identify, report and overcome all the barriers that could prevent Health Research Performing Organizations (HRPOs) from opening, sharing and FAIRifying their research data.

Then, FAIR4Health designed a workflow ^3^ to apply the FAIR principles to health research data, as well as to Electronic Health Record data, based on the FAIRification process of GO FAIR ^4^, but addressing the ethical, legal and technical aspects that health data include due to their sensitive nature by adding new steps in the workflow.

**Figure 1.**
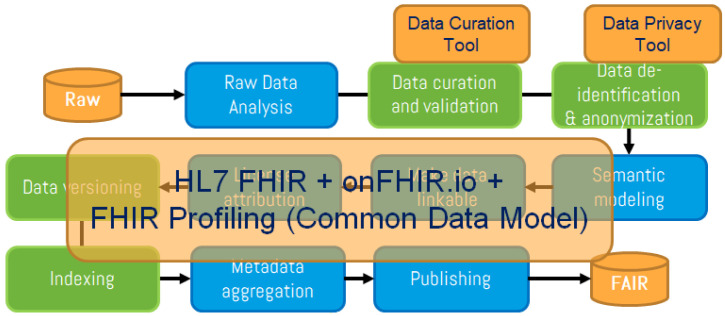
FAIR4Health workflow to apply FAIR principles in health research data. The FAIR4Health FAIRification workflow, based on the GO FAIR process (steps in blue), includes new steps (in green) to address the additional considerations for health data through curation, validation and anonymization of sensitive health data. Then, FAIRification tools, based on the use of the HL7 FHIR standard, were developed to obtain FAIR data from raw data.

As shown in Figure 1, new steps were included (in green) in the FAIR4Health FAIRification workflow to address these additional aspects through curation, validation and anonymization of sensitive health data. Adapted GO FAIR steps (in blue) define general actions for raw data analysis, license attribution, linking, semantic modeling, metadata management, and publishing to achieve FAIRness of existing (meta)data.

The requirements of health data were analysed in-depth, and FAIRification tools, based on the use of the HL7 FHIR standard, were developed to obtain FAIR data from raw data resulting from biomedical research.

FAIRification tools are standalone, desktop applications developed by the FAIR4Health project to perform “Data curation and validation” and “Data de-identification and anonymization” steps of the FAIRification Workflow in an easier way:

- Data Curation Tool ^5^ is a highly specialized Extract-Transform-Load tool that can extract data from relational databases and spreadsheets, apply user-defined transformations, and load the transformed resources into an HL7 FHIR repository.
- Data Privacy Tool ^6^ is responsible for handling the privacy challenges on sensitive health data by applying several data de-identification and anonymization techniques. After the curation process, the Data Manager uses the Data Privacy Tool to de-identify data before making it available to other systems/components as FAIR data. This tool reads and writes de-identified resources back to the HL7 FHIR repository.

**Figure 2.**
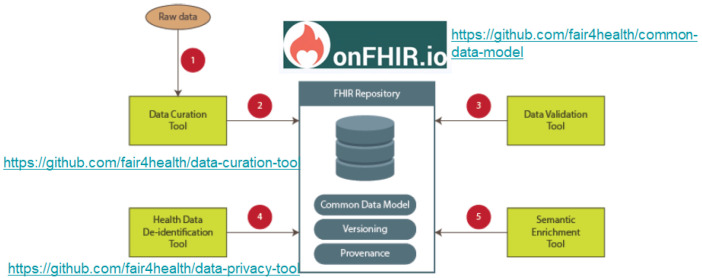
FAIR4Health architecture implementing the FAIRification Workflow for health data. At the core of architecture, an HL7 FHIR Repository acts as the health data repository. The FAIR4Health core architecture, which includes an FHIR Repository and is based on a Common Data Model, is an enabling factor for implementing the steps of the FAIR4Health FAIRification workflow in all aspects of FAIR principles.

Figure 2 shows the architecture implementing the FAIR4Health FAIRification Workflow for health data. In the core of architecture, an HL7 FHIR Repository acts as the health data repository. That way, the FAIR4Health core architecture, including an FHIR Repository and based on a Common Data Model, is an enabling factor for implementing the steps of the FAIRification workflow in all aspects of FAIR principles. In FAIR4Health, onFHIR.io was utilized as the HL7 FHIR Repository deployed within the agents.

On top of these, the FAIR4Health Platform was developed to apply a Privacy-Preserving Distributed Data Mining (PPDDM) framework enabling health research organizations to perform joint data mining operations without exposing any sensitive patient information to the outside world. In addition, the PPDDM Agent, which is responsible for running the data mining algorithms on top of the FAIRified data for the use cases defined by the user through the FAIR4Health Platform, was developed for training, validation and testing of models for the use cases defined. To achieve its objectives, the PPDDM Agent communicates with the onFHIR.io FHIR Repository within the data source boundaries, and the FAIR4Health Platform to exchange the results and predictive model information in a distributed manner.

**Figure 3.**
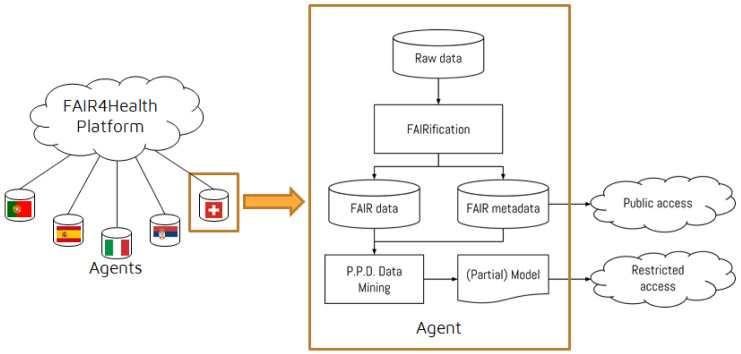
The overall architecture of the FAIR4Health solution. FAIR4Health Platform was developed to apply Privacy-Preserving Distributed Data Mining (PPDDM) models enabling health research organizations to perform joint data mining operations without exposing any sensitive patient information to the outside world. PPDDM Agents, which are responsible for running the data mining algorithms on top of the FAIRified data for the use cases defined by the user through the FAIR4Health Platform, were developed for training, validation and testing of models for the use cases defined. To achieve its objectives, the PPDDM Agents communicate with the onFHIR.io FHIR Repository within the data source boundaries, and the FAIR4Health Platform to exchange the results and predictive model information in a distributed manner.

The overall architecture of the FAIR4Health solution is shown in Figure 3.

## Results

The main objective of FAIR4Health was to facilitate and encourage the European Union Health Research community to FAIRify, share and reuse their datasets derived from publicly funded research initiatives through the demonstration of the potential impact that such a strategy has on health outcomes and health and social care research.

The FAIR4Health solution was validated with the two pathfinder case studies based on FAIRified data through the PPDDM framework.

1. Identification of multimorbidity patterns and polypharmacy correlation on the risk of mortality in elderly.
2. Early prediction service for 30-days readmission risk in patients with Chronic Obstructive Pulmonary Disease (COPD).

The goal of these case studies was to test the developed tools in the project. The prototypes were developed making use of federated machine learning methodologies and algorithms implemented upon the FAIR4Health Platform. First, each health research dataset was FAIRified using the FAIR4Health FAIRification tools. Then, the federated machine learning algorithms were trained and validated with retrospective datasets in both case studies. Finally, a prospective study was performed in the second use case to validate the developed model for prediction.

Concretely, the main goal of the pathfinder case study #1 was to analyze the impact of multimorbidity patterns and polypharmacy on the six-month mortality rate and cognitive impairment among elderly individuals in different health care settings. As a result, a multicentric retrospective observational study was designed in which data were collected from 5 different European cohorts. The population studied consisted of individuals aged 65 years or older with at least two chronic diseases. We used a frequent pattern tree association algorithm ^7^ implemented in the FAIR4Health Platform to identify the most frequent patterns in five different scenarios. The multimorbidity patterns obtained were consistent with previous studies ^8, 9^, which show the clinical potential of this method. We could also estimate a strong association between multimorbidity and polypharmacy and each of them with mortality.

COPD is one of the most prevalent chronic diseases. It has been associated with high morbidity and mortality and a high rate of readmission/rehospitalization and therefore associated with high healthcare costs. Thus, the main goal of the pathfinder case study #2 was to develop, validate and assess the accuracy of a clinical decision support tool for predicting 30-day readmission risk in patients suffering from COPD at discharge. In this line, the pathfinder case study #2 was composed of two phases to reach the main objective. The first one included a retrospective multicenter observational study, including the training and generation of prediction models in the FAIR4Health Platform. In the second phase, a prospective observational study with a 30-day follow-up was performed, from April 2021 to September 2021, to evaluate the accuracy of this tool by collecting data from a selected sample of subjects. The study population consisted of individuals aged 18 and older with a diagnosis of COPD who were admitted to the hospital for this disease. Finally, to assess the prediction risk accuracy associated with the early prediction service for 30-days readmission risk in COPD patients, predictions generated by the FAIR4Health Platform were compared with real-world data. The clinical assessment concluded that from 100 recruited patients, the prediction was correct in 87% of cases (that is, in real-life, the patient was readmitted and the algorithm predicted that there was early 30-days hospital readmission risk; or the patient was not readmitted and the algorithm predicted that there was not early 30-days hospital readmission risk).

Further details of the FAIR4Health pathfinder case studies can be found in the public report on the demonstrators’ performance ^10^.

## Conclusions/Discussion

FAIR4Health partners achieved the project’s objectives and the FAIR4Health use cases were successfully carried out through to the correct implementation of the technologies and performance of the complex FAIR4Health technical solution.

The main aim of the FAIR4Health project was to test the developed tools in the project: 1) application of FAIR principles in health research through the FAIR4Health FAIRification tools; 2) use of federated machine learning techniques; and 3) clinical, technical and functional validation of the FAIR4Health Platform and agents.

Therefore, FAIR4Health partners got positive conclusions from the FAIR4Health use cases. In both use cases, significant cross-cutting data-related issues and challenges were identified and addressed. The task to extract data from EHRs and other kinds of healthcare sources aligning this extraction with a FAIR4Health Common Data Model was not trivial and required a lot of conceptual and technical efforts, because: (i) complexity of the raw data (the source EHRs are commonly very complex including information in several tables in the source databases); (ii) free text used in some fields in the raw data sources; and (iii) differences between the type of the raw data sources. To address the complexity of the raw data, each health research organization from different countries that participated in data extraction involved colleagues who were experts in each source data model. To address the information in free text fields, Natural Language Processing (NLP) techniques were assessed, and finally, in some cases, manual NLP to extract structured information from unstructured information was performed. Due to the differences in the raw data sources, each raw dataset had to be analyzed in depth in collaboration between the clinical partners and the technical partners. This involved determining the required configuration of the FAIR4Health solution to enable FAIRification of all raw data. Finally, coordinated federated machine learning models were created using all sources.

It is relevant to add other significant conclusions here, related to the application of the FAIR principles in health research:

- Implementation of FAIR principles allowed us to use larger and more heterogeneous datasets in FAIR4Health, increasing the variability of the data, the size of the datasets, and finally, more comprehensive and reliable results/outputs, compared to specific research studies without applying FAIR.
- We could reuse FAIR datasets from other clinical organizations in a secure way, ensuring compliance with General Data Protection Regulation (GDPR), and we could use the clinical datasets in the federated machine learning models. In the FAIR4Health project, we could also consider demographic, environmental, clinical and social information. We achieved greater variability of datasets and inclusion of more variables, compared to research where FAIR datasets are not reused.
- We obtained an increase in the scope of the research and improvements in health research, facilitating the discovery of scientific knowledge through data sharing and data reuse. Likewise, FAIR data reuse provided savings in data collection where much effort is currently invested.
- The implementation of FAIR principles facilitated the reproducibility of the study and access to large volumes of data to make the research more robust. We obtained the increase in secondary use of datasets once FAIR policies were implemented, related to the publication and sharing of FAIR datasets.

## Data and software availability

### Underlying data

No data are associated with this article.

### Extended data

Along with the FAIR4Health software, FAIR metadata related to the FAIRified datasets generated in the FAIRification process, is published in the FAIR4Health GitHub. This is available to the scientific community, and the FAIR4Health consortium continues assessing the possibilities to open publish these metadata in other public repositories. Further information: https://github.com/fair4health/.

Data are available under the terms of the Creative Commons Zero "No rights reserved" data waiver (CC0 1.0 Public domain dedication).

## References

[1] Wilkinson MD Dumontier M Aalbersberg IJ : The FAIR Guiding Principles for scientific data management and stewardship. *Sci Data.* 2016;3:160018. 10.1038/sdata.2016.18 26978244 PMC4792175

[2] European Commission: Cost of not having FAIR research data - Cost-Benefit analysis for FAIR research data.2018. Reference Source

[3] FAIR4Health Guidelines for implementing FAIR open data policy in health research. Reference Source

[4] Sinaci AA Núñez-Benjumea FJ Gencturk M : From Raw Data to FAIR Data: The FAIRification Workflow for Health Research. *Methods Inf Med.* 2020;59(S 01):e21–e32. 10.1055/s-0040-1713684 32620019

[5] FAIR4Health Data Curation Tool. Reference Source 10.3233/SHTI210110 34042695

[6] FAIR4Health Data Privacy Tool. Reference Source

[7] Han J Pei J Yin Y : Mining frequent patterns without candidate generation. *ACM sigmod record.* 2000;29(2):1–12. 10.1145/335191.335372

[8] Poblador-Plou B Calderón-Larrañaga A Marta-Moreno J : Comorbidity of dementia: a cross-sectional study of primary care older patients. *BMC Psychiatry.* 2014;14(1):84. 10.1186/1471-244X-14-84 24645776 PMC3994526

[9] Prados-Torres A Calderón-Larrañaga A Hancco-Saavedra J : Multimorbidity patterns: a systematic review. *J Clin Epidemiol.* 2014;67(3):254–266. 10.1016/j.jclinepi.2013.09.021 24472295

[10] FAIR4Health Report on the demonstrators performance. Reference Source

