# Supplementary material for: FAIR4Health: Findable, Accessible, Interoperable and Reusable data to foster Health Research
Source: Open Res Eur. 2022 May 31;2:34. Originally published 2022 Mar 9. [Version 2] doi: 10.12688/openreseurope.14349.2 (PMC10446092; doi:10.12688/openreseurope.14349.2)
Supplement: Peer review history

# Peer review: Reviewer response for version 2

**DOI:** 10.21956/openreseurope.16044.r29410

**Published:** 2022-06-24

**Authors:** Anupama Gururaj

**Affiliations:** 1 National Institute of Allergy and Infectious Diseases, National Institutes of Health, Rockville, MD, USA

The authors have appropriately addressed the concerns raised previously.

Is the study design appropriate and does the work have academic merit?

Yes

Is the work clearly and accurately presented and does it cite the current literature?

Partly

If applicable, is the statistical analysis and its interpretation appropriate?

Not applicable

Are all the source data underlying the results available to ensure full reproducibility?

Yes

Are the conclusions drawn adequately supported by the results?

Partly

Are sufficient details of methods and analysis provided to allow replication by others?

Yes

Reviewer Expertise:

Biomedical data sharing and management, clinical informatics

I confirm that I have read this submission and believe that I have an appropriate level of expertise to confirm that it is of an acceptable scientific standard.

# Response

**Published:** 2022-07-27

**Authors:** Celia Alvarez

**Affiliations:** Biomedicine Institute of Sevilla - Andalusian Health Service, Spain

Thank you very much for reviewing our article. All the best.

# Peer review: Reviewer response for version 1

**DOI:** 10.21956/openreseurope.15486.r28796

**Published:** 2022-04-19

**Authors:** Salvador Capella-Gutierrez

**Affiliations:** 1 Department of Life Sciences, Barcelona Supercomputing Center (BSC), Barcelona, Spain

Alvarez-Romero and colleagues provided a fascinating article on how FAIR data in health research can benefit researchers and patients. This effort is part of the recently finished FAIR4Health project and illustrates its progress.

 I have to congratulate the authors on the extended FAIRification process for health research data. I appreciate the data versioning as an essential step toward reproducibility of any result.

 I have some comments aiming to foster the discussion and clarify some aspects of this manuscript:

1. Is there any specific reason to focus on HL7 FHIR as an interoperability standard across participating centres? Despite HL7 FHIR, I'd expect at least a mention of other standards to facilitate health-related research, e.g. OMOP. This aspect is especially relevant when working with observational studies, e.g. cohort-based research. Thus, the interesting point is to know how extensible this work is and the technical implications of making such an effort.
2. Can you provide additional details on the privacy-preserving mechanisms? If it has already been published somewhere else, a couple of sentences summarising it with the reference should be enough.
3. I wonder about the use of onFHIR platform. I assume it has been developed by one of the FAIR4Health partners. Then it makes sense to use it. I appreciate the fact of using open-source software. Can similar tools/platforms replace it? How interoperable are the outputs of this platform? I'm thinking of interested parties whiling to use/leverageFAIR4Health outcomes having their solutions.
4. I'd suggest delineating/introducing the use-cases earlier in the text.
5. I find figure #3 very informative. However, it led me to ask myself about potential mechanisms for accessing data produced in the consortium. If there is any formal mechanism to access or request access to those datasets, I think they can be included in this figure.
6. Looking at use-case #1, you mentioned that implemented algorithms are available as part of the platform in the text. Perhaps you can include a link to it, e.g. a specific repository in the FAIR4Health GitHub Organization.
7.  Still looking at use-case #1, you mentioned: "... a strong association between multimorbidity and polypharmacy and each of them with mortality." Have you performed any statistical analysis here? Perhaps it is good to include them as part of the use-case #1 discussion.
8. I like the description of use-case #2 and appreciate that there is an extensive report (68 pages) describing it. However, readers would appreciate a short explanation of the main findings for this use-case. Otherwise, it seems a bit disconnected.
9. You have briefly discussed the possibilities of using FAIR data for distributed ML across different sites. Can you explain the minimal computational requirements for carrying on these analyses? I think it is essential for readers to realise that it is not enough to have FAIR data at their sites but also the computational capabilities to conduct such analyses.
10. I like the description of the efforts to FAIRify health research data. However, I'm missing the language axis when using NLP technologies. NLP models and resources are language-dependent, which means that the final results are partially affected by them. As you are working with data from 5 different European countries, can you share your experiences on these aspects?

Looking forward to your comments on those aspects.

Is the study design appropriate and does the work have academic merit?

Yes

Is the work clearly and accurately presented and does it cite the current literature?

Yes

If applicable, is the statistical analysis and its interpretation appropriate?

Partly

Are all the source data underlying the results available to ensure full reproducibility?

Yes

Are the conclusions drawn adequately supported by the results?

Yes

Are sufficient details of methods and analysis provided to allow replication by others?

Partly

Reviewer Expertise:

FAIR principles

I confirm that I have read this submission and believe that I have an appropriate level of expertise to confirm that it is of an acceptable scientific standard, however I have significant reservations, as outlined above.

# Response

**Published:** 2022-05-23

**Authors:** Celia Alvarez

**Affiliations:** Biomedicine Institute of Sevilla - Andalusian Health Service, Spain

Dear reviewer, All comments and recommendations have been addressed. Improvements and expanded descriptions of some parts of the article have been included in the new version, along with contributions suggested by the other reviewers.

 Thank you very much for the opportunity to improve the manuscript and for all the comments, very timely to improve the scientific quality. Best regards.

# Peer review: Reviewer response for version 1

**DOI:** 10.21956/openreseurope.15486.r28777

**Published:** 2022-04-08

**Authors:** Gary Saunders

**Affiliations:** 1 EATRIS ERIC, Amsterdam, The Netherlands

This is a strong brief report of two use cases utilising the FAIR4Health project solution in addressing two different use cases of health data demonstrating the benefits in the application of the FAIR principles to such data that result in strong conclusions. Furthermore, the authors have published and made available all software that is referenced in the report so that it is available to the community.

 I support the publication of this report, and have only two minor corrective suggestions:

1. Please can the authors include the total number of individuals that were analysed in use case 1? It would also be good to have this number broken down to the 5 distinct populations, however this may not be possible due to privacy concerns. As the use case discusses the application of federated ML it would be good to have some indication of sample size that was used in the analysis.
2. The NLP techniques that are discussed in the conclusion should be described earlier in the manuscript. There is some nice text in the discussion section not only of the NLP techniques but also of the complex application of the FAIR4Health Common Data Model to the use cases discussed in the report. It would be good to have this properly described in the Methods section of the manuscript as not only will this aid transparency in the processes, and therefore reproducibility of the results described, but also aid the community in the potential application of the CDM to future datasets.

In summary, this is a nice, neat and concise manuscript describing the work in an easy-to-read, follow, and digest manner.

Is the study design appropriate and does the work have academic merit?

Yes

Is the work clearly and accurately presented and does it cite the current literature?

Yes

If applicable, is the statistical analysis and its interpretation appropriate?

Partly

Are all the source data underlying the results available to ensure full reproducibility?

No source data required

Are the conclusions drawn adequately supported by the results?

Yes

Are sufficient details of methods and analysis provided to allow replication by others?

Partly

Reviewer Expertise:

FAIR data, big data management, federated data access and analysis, health care data.

I confirm that I have read this submission and believe that I have an appropriate level of expertise to confirm that it is of an acceptable scientific standard, however I have significant reservations, as outlined above.

# Peer review: Reviewer response for version 1

**DOI:** 10.21956/openreseurope.15486.r28776

**Published:** 2022-04-04

**Authors:** Anupama Gururaj

**Affiliations:** 1 National Institute of Allergy and Infectious Diseases, National Institutes of Health, Rockville, MD, USA

This paper describes a project focusing on the need to make healthcare data FAIR and reusable in the European Union context. The study addresses an emerging research need within the healthcare system and will be of interest to clinical research personnel in various healthcare settings. This team has performed two pilot studies that outline a general framework that can be adopted by other systems. The manuscript is concise and provides an overview of the project and project outcomes including tools developed and use cases addressed.

 Major Criticisms:

1. In the abstract, the authors mentioned access to certified FAIR datasets. It is not clear in the rest of the manuscript how and who has provided such certification to the metadata of the two datasets that are shared. Please provide further details or remove mention of certification.
2. In parts, the paper overstates some aspects of the methods and conclusions. As an example, in the methods section, it is stated that the FAIR4Health provides an overview of all considerations to overcome all barriers to open research data sharing by HRPOs. Likewise, in the conclusions/discussions section, the authors state that they have seen an increase in secondary use of the datasets without providing any usage metrics. My recommendation is to tone down the absolute statements.
3. It would be useful to the reader to get a better understanding of the Common Data Model that is used to harmonize the data from the various sources. Please provide a description of the model in the paper.
4. Other large-scale efforts such as OHDSI are community-led and have leveraged distributed analytics for answering scientific questions. Please compare and contrast the FAIR4Health initiative to such efforts in the discussion.
5. It would be useful to the community to hear about lessons learnt and challenges that have not yet been solved through the life of the project. Also, please comment on the scalability of the approach since a lot of manual effort and coordination was a part of the project.

Minor comments:

1. Some of the sentences (such as the starting sentence of the second paragraph of the introduction (The FAIR guiding principles…) are very long and make the paper hard to read. It is recommended to simplify or split these sentences.
2. Figure 1 is hard to read and follow since there are overlapping boxes. Please fix the figure to increase clarity.

Is the study design appropriate and does the work have academic merit?

Yes

Is the work clearly and accurately presented and does it cite the current literature?

Partly

If applicable, is the statistical analysis and its interpretation appropriate?

Not applicable

Are all the source data underlying the results available to ensure full reproducibility?

Yes

Are the conclusions drawn adequately supported by the results?

Partly

Are sufficient details of methods and analysis provided to allow replication by others?

Yes

Reviewer Expertise:

Biomedical data sharing and management, clinical informatics

I confirm that I have read this submission and believe that I have an appropriate level of expertise to confirm that it is of an acceptable scientific standard, however I have significant reservations, as outlined above.
